# Reproductive Potential Impacts Body Maintenance Parameters and Global DNA Methylation in Honeybee Workers (*Apis mellifera* L.)

**DOI:** 10.3390/insects12111021

**Published:** 2021-11-12

**Authors:** Jerzy Paleolog, Karolina Kuszewska, Michał Woyciechowski, Aneta Strachecka

**Affiliations:** 1 Department of Invertebrate Ecophysiology and Experimental Biology, University of Life Sciences in Lublin, 20-950 Lublin, Poland; jerzy.paleolog@up.lublin.pl; 2 Institute of Environmental Sciences, Jagiellonian University, 30-387 Krakow, Poland; k.kuszewska@gmail.com (K.K.); michal.woyciechowski@uj.edu.pl (M.W.)

**Keywords:** *Apis mellifera*, DNA methylation, lifespan, nosemosis, phenotypic plasticity, rebel, workers

## Abstract

**Simple Summary:**

The queens and sterile workers arise from genetically identical eggs but as imagoes, they differ in their life span, DNA methylation, and their functions. In the absence of the queen, the larvae develop into rebels, i.e., workers with increased reproductive potential. We assumed that since rebels are similar to the queen in many anatomical and behavioral features, they live longer and have lower levels of global DNA methylation, even when infected, e.g., by *Nosema* spp. Rebels always lived longer in comparison in normal workers and unexpectedly extended longevity of normal workers when they were together, similarly as the presence of a queen did. Rebels became infected more easily but tolerated the infection better. They also had lower level of global DNA methylation than normal workers. These features expand possibilities of the use of honeybees as a model for studies on senescence, nosemosis, eusocial evolution, and epigenetics.

**Abstract:**

The widely accepted hypothesis in life history evolution about the trade-off between fecundity and longevity is not confirmed by long-living and highly fecund queens in eusocial insects. The fact that the queens and facultatively sterile workers usually arise from genetically identical eggs but differ in DNA methylation makes them a good model for studies on senescence, eusocial evolution, and epigenetics. Therefore, honeybees seem to be especially useful here because of long living rebel-workers (RW) with high reproductive potential recently described. Longevity, ovariole number, nosema tolerance, and global DNA methylation have been assayed in normal workers (NW) versus RW in hives and cages. RW always lived longer than NW and unexpectedly extended longevity of NW when they were together, similarly as the presence of a queen did. RW lived longer despite the fact that they had higher Nosema spore load; surprisingly they became infected more easily but tolerated the infection better. Global DNA methylation increased with age, being lower in RW than in NW. Therefore, RW are queen-like considering global DNA methylation and the link between fecundity, longevity, and body maintenance. Presented features of RW expands possibilities of the use of honeybees as a model for studies on senescence, nosemosis, eusocial evolution, and epigenetics.

## 1. Introduction

The traditional model describes the trade-off between fecundity and longevity [1], but a fertile queen of perennial social insects like in honeybees has usually a much longer lifespan than do the facultatively sterile workers. The older age is usually linked with the decrease of the body maintenance abilities; therefore, efficient body maintenance mechanisms are needed for long-living reproductive females [2,3]. This has been proved in the recent study of Paleolog et al. [4], as the antioxidative defense of the old queens was still efficient but not of the sterile foragers (older workers). Consequently, positive complex links among the reproductive potential, longevity, and body maintenance could be evolutionarily shaped not only in the queens but also in those specific workers that have a higher reproductive potential [5].

The fact that honeybee queens and workers with similar genotypic backgrounds distinctly differ in longevity, reproduction, and body maintenance points out the existence of a huge phenotypic plasticity in members of one colony. Therefore, honeybees have been widely used in studies on senescence or on the mechanisms of the evolutional ecology [6,7,8]. In this context, more attention should be paid to the recently described so-called rebel workers (RW), which are the workers with high reproductive potential compared to typical facultatively sterile normal workers (NW) described by Woyciechowski and Kuszewska [9]. RW arise from larvae reared in the absence of a queen and they are more queen-like than NW, not only because they live longer and have more ovarioles staying active even in queenright colony, but their mandibular and Dufour’s glands are more developed, whereas hypopharyngeal glands are underdeveloped [9,10,11,12]. That is why we expect that the presence of the queen-like RW can affect longevity of NW in a similar manner as the presence of a queen can do.

RW ought to be also more tolerant to nosemosis, which is one of the major threats to western honeybees [13], i.e., they ought to have better body maintenance, as the tolerance is the essential component of it, precisely due to this threat. To the best of our knowledge, there is no information about it. Therefore, we decided not to plan a separate experiment, but to use the *Nosema* infected bees from an already infected apiary. *Nosema* infection affects the health of honeybee hosts via disturbance of hormones, reduction of lifespan, degeneration of midgut epithelial cells, energetic stress, cell apoptosis inhibition, and immunity suppression [13] which is manifested by a change in gene expression and in the epigenome, particularly in DNA methylation [14,15].

Epigenetic studies involving comparing global DNA methylation in NW versus more queen-like RW could also be promising. Knowledge about this matter does not exist either. It is important because global DNA methylation is believed to be involved in shaping the phenotypic differences between queens and workers [14,15,16]; therefore, we expect that they might also affect the differences between RW and NW. On the other hand, the level of the global DNA methylation increases with their age [17,18]. This, together with the finding that the increase of the workers’ lifespans is connected with the decrease of their global DNA methylation [19,20] and that the artificial genome demethylation increases the worker longevity [21] suggest that longer living RW can be used as a useful model to clarify the role of the global DNA methylation in the bee female phenotype plasticity and ageing, and more generally, in epigenetic aspects of eusocial evolution.

Considering that honeybee females’ lifespan or life expectancy depends on their reproductive potential and its function in a society, as well on the presence of the nosema infection, we exanimated longevity, nosema tolerance, and global DNA methylation in both free flying (hives) and caged (laboratory) RW versus NW. This approach also helps to expand possibilities of the use of honeybees as a model organism for gerontology, social evolution, and epigenetic studies.

## 2. Materials and Methods

Studies were performed at Life Sciences University in Lublin, Poland (51°225 N–22°635 E) at the isolated apiary where colonies appeared to be infected both with the *Nosema apis* and *N. ceranae* when flying bees were captured and assayed (compare Sulborska et al. [22]). The genetic analyse of bees used in this study confirmed that they were infected by these both parasites.

### 2.1. Preliminary Procedures and Hive Tests

**Figure 1:**
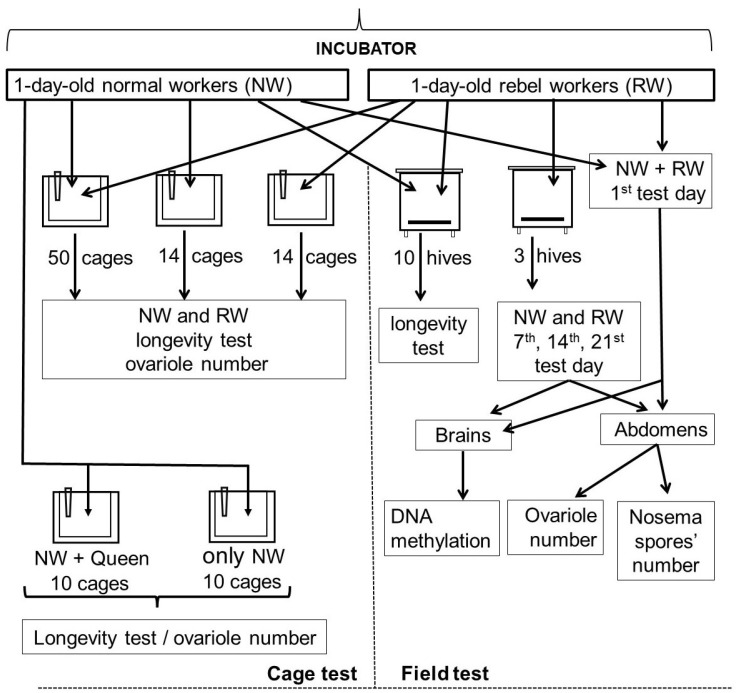
Schemes of the cage and field (in hives) experiments.

Ten honeybee colonies headed by sister *Apis mellifera carnica* queens (simultaneously, naturally inseminated at the same bee yard) kept in fully populated two-super Dadant-Blatt hives were used as adult bee rearing colonies for further field and cage experiments. Each queen was restricted within a queen-excluder cage, containing two combs (C1 and C2), to lay eggs for 24 h. After three days, each colony was divided into two equal subunits. Each of them contained workers and brood and had its own entrance. The first, queenright subunit containing C1, was used to rear NW, whereas the second, queenless subunit containing C2, was used to rear RW (see method used by Woyciechowski and Kuszewska [9]). After the brood in C1 and C2 had been sealed, the subunits were reconnected. Shortly before the worker emergence, all C1 and all C2 were placed in the separate incubator units (34.5 °C, RH 60%) and both the emerging, one-day-old NW (from C1) and one-day-old RW (from C2) were frozen at −40 °C for assessment of global DNA methylation, ovariole number, and *Nosema* spore number, or intended for further cage tests, or paint-marked with the specific cohort colors and introduced to 13 colonies populating 6-frame (251 mm × 159 mm) mini-plus hives; 500 RW and 500 NW per hive (Figure 1). Three colonies were used for determining the global DNA methylation, ovariole number, and *Nosema* spore number. Ten RW and 10 NW were captured from each of these colonies on the 7th, 14th, and 21st day and frozen at −40 °C for further dissection. The remaining 10 colonies were intended for the longevity tests on the following way. Both RW and NW were counted on the combs outside the foraging activity to assess their losses/mortality every 5th–7th test day depending on the weather in each of these 10 colonies. The longevity and *Nosema* spore number tests were then repeated (repetition 2) using, respectively, 4 and 4 hives, applying the same protocol.

### 2.2. Cage Tests

One-day-old NW and RW (Figure 1) taken from the incubator were also used for the longevity and *Nosema* spore number cage tests that were performed simultaneously with the hive tests. The cage test conditions were as follows: temp. 25 °C. RH 65%. Wooden cages (12 cm × 12 cm × 4 cm) with one movable glass wall, ventilation and opening slots, and feeders supplied with sugar syrup were used. One experimental and two reference groups were established. Forty-three cages, each containing the mix of 25 RW and 25 NW constituted the experimental group (for the cage bee population size compare Bosua et al. [23]). The first reference group consisted of 30 cages: 15 cages containing only the RW (40 in each) and 15 cages containing only NW (40 in each). The second reference group consisted of 20 cages; 10 cages containing 50 NW in each and 10 cages containing 50 NW accompanied by the queen in each. Dead workers were removed and counted in each of the cage at 2-day intervals (mortality) and then frozen at −40 °C for further dissection to assess the *Nosema* spore number and number of ovarioles.

### 2.3. Laboratory Protocols

Brains were dissected from each RW and NW captured from the three hives (Figure 1) and frozen (−40 °C). Then, digestive tracks were dissected from the abdomens of these RWs and NWs and used for determining the *Nosema* spore number [24] and type of *Nosema* spores. Subsequently, the abdomens were cut lengthwise and the ovarioles in two ovaries were counted [9] using Stereo Zoom Microscope: Olympus SZX16 (Olympus Corporation, Tokyo, Japan); Camera: Olympus DP72 (Olympus Corporation, Tokyo, Japan). All ovarioles were stained with Giemsa reagent (Sigma-Aldrich, Poznan, Poland) 10 s before their examination.

After thawing, DNA was individually extracted from each bee’s brain using the DNeasy Blood and Tissue Kit (Qiagen, Hilden, Germany), stored at −25 °C, and later used to determine global DNA methylation applying the Imprint Methylated DNA Quantification Kit (MDQ1-96RXN-Sigma, Ronkonkoma, NY, USA). The manufacturers’ instructions were followed during the DNA extraction, as well as in the calculation of the global DNA methylation percentage and weight (ng).

To determine the type of *Nosema* spores, DNA was isolated from the RW and NW digestive tracts using the DNeasy Blood and Tissue Kit (Qiagen Polska, Wroclaw, Poland) according to the manufacturer’s instructions. Subsequently, each of the DNA samples was used as a template for detection of *N. apis* and *N. ceranae* 16S rDNA by PCR with Nosema-specific primers (Genomed, Warsaw, Poland): 321-APIS for *N. apis* and 218-MITOC for *N. ceranae* [25]. All the bees had *N. ceranae* and *N. apis*.

### 2.4. Statistical Analysis

The longevity of workers was estimate using the Kaplan–Meier estimator and next to compare different Kaplan–Meier curves the Log-Rank tests were done (in our research all individuals were dead at the end of experiment and we have no censor data, however Log-Rank survival analysis gives the opportunity to test censored data). Numbers of *Nosema* spores were tested by multiple one-way ANOVAs; factor: the worker phenotype (RW/NW). For each age group/collection (dependent variables), these characteristics were compared between RW and NW. The ovariole numbers, global DNA methylation levels, and methylated cytosines weights were tested by three-way ANOVAs. The phenotypes of workers (NW/RW) and days of life were fixed factors while colony was a random factor. Statistically significant ANOVA results were followed by multiple comparisons using the post-hoc Tukey HSD test, with *p* < 0.05 considered significant. All calculations for all experiments were performed with STATISTICA version 12.0 (Stat Soft Inc., Tulsa, OK, USA).

## 3. Results

**Figure 2:**
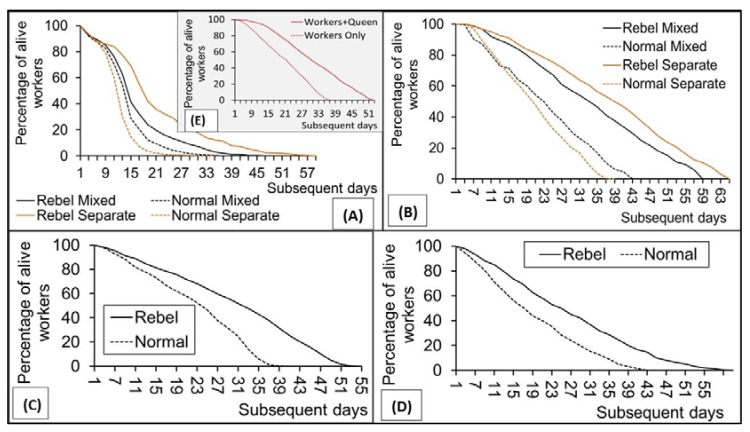
Survival of the rebel and normal workers kept in cages (**A**,**B**—repetition 1 and 2) and survival of the rebel and normal workers kept in hives (**C**,**D**—repetition 1 and 2). Survival of the normal workers kept in the cages with queens or without queens (**E**). Explanations: Rebel Mixed and Normal Mixed—cages contained both rebel and normal workers (RW + NW). Rebel Separate and Normal Separate—the rebel or normal workers were kept in the separate cages.

RW lived longer than NW, regardless of whether they were kept in the hives (Repetition 1: Log-Rank test Z = −31.09, *p* < 0.001; Repetition 2: Log-Rank test Z = 19.18, *p* < 0.001) or in cage, both in the first (Repetition 1: Mix bees Log-Rank test Z = 10.09, *p* < 0.001; Repetition 1: Separate bees Log-Rank test Z = 19.41, *p* < 0.001) and in the second repetition of the longevity test (Repetition 2: Mix bees Log-Rank test Z = 11.43, *p* < 0.001; Repetition 2: Separate bees Log-Rank test Z = 20.41, *p* < 0.001; Figure 2A–D). Importantly, NW lived longer and RW shorter in cages containing their mix than when NW and RW were kept in separate cages ((NW-Repetition 1: Mix bees Log-Rank test Z = −8.78, *p* < 0.001; RW-Repetition 1: Separate bees Log-Rank test Z = 14.35, *p* < 0.001; NW-Repetition 2: Mix bees Log-Rank test Z = −4.30, *p* < 0.001; RW-Repetition 2: Separate bees Log-Rank test Z = 5.88, *p* < 0.001). All differences were confirmed using Kaplan–Meier estimation and next the Log-Rank test to compare the different Kaplan–Meier curves (see Figure 2A–D). Other analyses showed that presence of a queen (Figure 2E) also expanded longevity of NW in cages (Log-Rank test Z = −13.55, *p* < 0.001).

**Figure 3:**
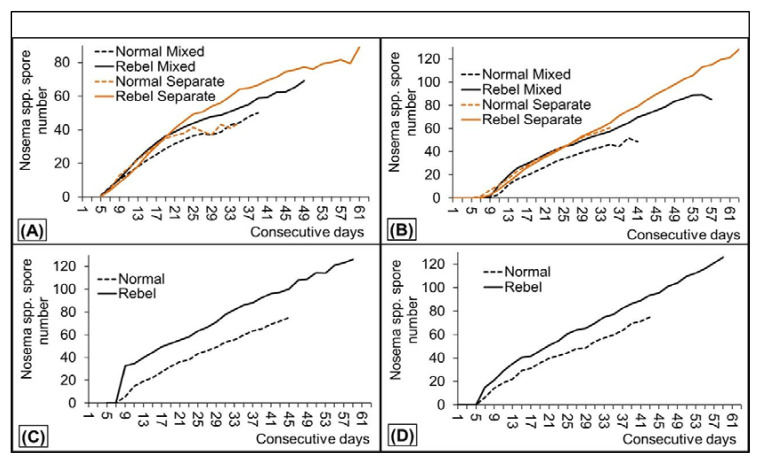
*Nosema* spp. spore number in 1 µL in the macerates of abdomens of rebel versus normal workers kept in cages (**A**,**B**—repetition 1 and 2) or kept in the hives (**C**,**D**—repetition 1 and 2). Explanations: Rebel—rebel workers; Normal—normal workers. All necessary statistics are included in Supplementary Materials (Table S1).

RWs lived longer and were more infected with *Nosema* spores (Figure 3A,B) in cages containing a mix of RWs and NWs. They had higher spore loads, both in repetition 1 and 2. For instance, F = 4.7911 and *p* = 0.03138 on the 11th day and F = 41.911 and *p* = 0.00000 on the 19th day of life in repetition 1 or F = 16.626 and *p* = 0.00034 on the 11th day and F = 14.458 and *p* = 0.00071 on the 31st day of life in repetition 2. RWs also had higher spore loads than NWs in both repetitions in the hives (Figure 3C,D). However, the differences were not significant in repetition 2; *p* ranged from *p* = 0.00649 to *p* = 0.05005 in repetition 1, whereas it varied from *p* = 0.29881 to *p* = 0.65816 in repetition 2. Importantly, RWs had higher spore loads when kept together with NWs in one cage. Thus, it could not be a sampling coincidence due to mutual spore transmission between them in one cage. This, however, was not so evident when RWs or NWs were kept in separate cages, as results obtained in the two test repetitions were different. All the necessary statistics and comparisons are included in Supplementary Materials (Table S1).

**Figure 4:**
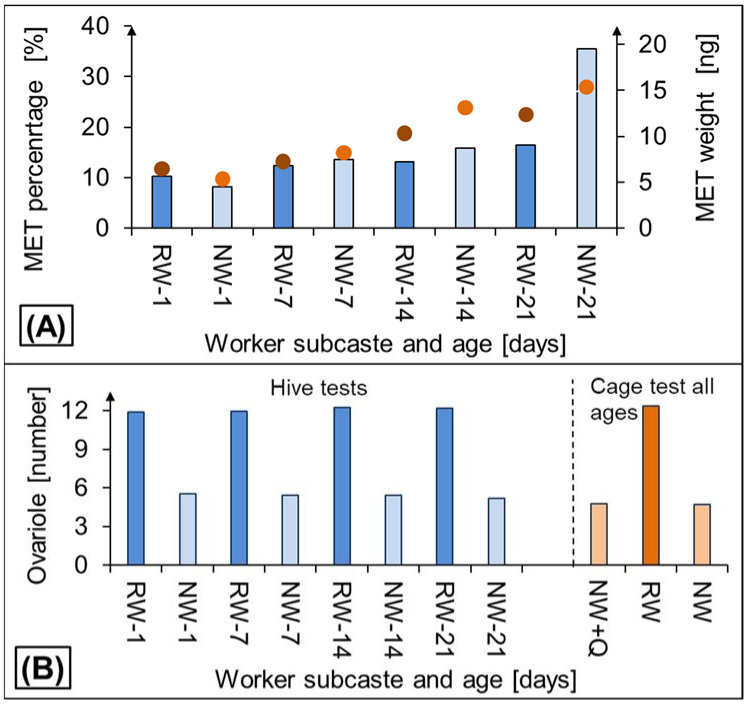
(**A**) Global methylation level (MET) in rebel (RW) versus normal (NW) workers expressed (three-way ANOVA: colony F_2,216_ = 1.41; *p* = 0.246, phenotype: F_1,2_ = 2291.77; *p* < 0.001; day of life: F_3,6_ = 4358.95; *p* < 0.001; Tukey test for day of life *p* < 0.001 for all comparison) by bars and the methylated cytosines weight expressed by circles (three-way ANOVA: colony F_2,216_ = 1.00; *p* = 0.379, phenotype: F_1,2_ = 955.00; *p* < 0.001; day of life: F_3,6_ = 6429.7 *p* < 0.001; Tukey test for day of life *p* < 0.001 for all comparison). (**B**) Ovariole numbers in RW and NW kept in hives or cages. RW—rebel workers; NW—normal workers (three-way ANOVA: colony F_2,216_ = 8.56; *p* > 0.379, phenotype: F_1,2_ = 8554.68; *p* < 0.001; day of life: F_3,6_ = 0.13 *p* > 0.05); when RW or NW are followed by the worker age, e.g., RW−7 or NW−21 it means 7-day-old rebels or 21-day-old normal workers, respectively; when NW are followed by +Q, workers were accompanied with queen. More details are provided in Supplementary Materials (Table S2).

Global DNA methylation increases with age both in RW and NW. Except for the first day of the worker’s life, it was always lower in RW than in NW, particularly in 21-day-old bees (Figure 4A).

The ovariole number was almost three-fold higher in RW than in NW (Figure 4B) regardless of the worker age and their experimental environment. Standard deviations ranged with the age from 0.245 to 0.298 for RW or from 0.241 to 0.326 for NW when assayed in hives, but surprisingly, this statistic was 6.5 times lower in the cages (0.041 for RW or 0.041 for NW). Additional information is given in Supplementary Materials (Table S2).

## 4. Discussion

The marked and steady difference between the ovariole numbers in NW and RW considered in light of the previous research [9,10] proved that we assayed RW, that are a separate worker phenotype with a high reproductive potential and that this potential is distinctly higher than that in NW.

Our cage and hive experiments confirmed the findings of Kuszewska et al. [10] and Kennedy et al. [5] that workers with higher reproductive potential, like RW, live longer than NW. This, together with the fact that NW and RW can develop under the natural hive conditions [9], may expand possibilities of using honeybees for studies on different aspects of senescence (compare Tyler et al. [26]).

RW appeared more queen-like and NW more queen-dislike at the older age, as the difference between RW and NW survival was smaller in the first week of their life. This corresponds with the finding that NW antioxidative defense was more queen-like during the first week of their life but appeared to be different than those in the queens at the forager stage; old workers [4].

Our predicted finding that there may exist relations between NW and RW which affect their lifespan can shed new light on life expectancy issues and the aging process in general, as the presence of RW expanded NW longevity, whereas presence of NW shortened the longevity of RW in our cage experiments. We also confirmed the earlier findings that the presence of a queen expanded longevity of the caged NW [27,28], showing, however, that the queen does it to a greater extent than RW. Therefore, RW had to have a queen-like effect on NW longevity when they dwell together. Past research indicated that the longevity of workers decreased with prolonged queenlessness in the field experiment [29]. On the other hand, queenlessness expanded the worker longevity and reproductivity of bees [5,9,10] and ants [30], but only when the queens were absent at the worker larval stage. Hence, the presence of a queen affects longevity of workers in different ways depending on whether the queen is present at their larval or imago stage. The previous results showing that RW have larger mandibular glands producing QMPs [31] and that RW combine many features of the fat bodies characteristic of both queens and workers [32] confirm that RW may exert a queen-like effect on NW longevity when these two worker types dwell together at the imago stage. This finding markedly expands the knowledge about the workers with the higher reproductive potential.

*N. ceranae*, microsporidian causing nosemosis, is one of the major, current threats to honeybees. Our bees, sampled from the colonies originated from the infected apiary [22], consequently appeared to be infected by this parasite, which has been finally confirmed by the genetic test. The microsporidian has deleterious effects on honeybee survival, immunity, antioxidative barriers, and detoxicate defense [13,33,34,35,36] being the essential component of the apian body maintenance.

Queens have more antimicrobial peptides than workers [37]. The response of these peptides was opposite in queens and workers when they were infected with *N. ceranae*, as the parasite downregulated the immunity genes in workers but upregulated them in queens [38,39]. Vitellogenin is also involved in honeybee immune and biochemical defense [7]. Its titers increased in queens infected with *N. ceranae* [40], whereas genes determining the titers were downregulated in workers infected with this pathogen [35,38]. If nosema tolerance in RW was queen-like, they would be more resistant to the *Nosema* spores’ penetration, and then, would deal better with the developing nosemosis. Consequently, the infected RW ought to live longer and have smaller spore load than NW. Our results only partly fit this assumption, as RW lived longer than NW, but at the same time they had higher *Nosema* spore loads. In other words, although RW coped better with the nosemosis during its course, they seemed to be more sensitive to the microsporidian spore penetration. This conclusion seems to be a little bit surprising and speculative but the phenomenon happened both in hives and cages, in which RW and NW lived together and were exposed to mutual spore transmission. This rules out the randomness of this surprising phenomenon. On the other hand, the observation is new and very interesting, and therefore, it should be presented to readers for further investigations to answer whether the nosema tolerance may consist of two different, independent components. The first is resistance of the worker midgut cells against penetration of the spores, which depends on cytoplasm transferrin receptor. The second is the nosemosis tolerance in already infected workers, which depends mainly on the availability of the host energy resources, as the parasite hijacks energy from its cells “using” its mitochondria/ATP; an elevated carbohydrate turnover rate was noted in tolerant bees [13]. Our results may suggest that the first component is probably more efficient in NW, whereas the second, in RW. Therefore, the use of RW versus NW for further studies on mechanisms of the nosema tolerance might be considered, as the knowledge about these mechanisms is still limited [13,39,41]. It is puzzling that the differences between the nosema tolerance in RW and NW were not so evident when RW and NW were kept in separate cages. This is another question about interrelations between RW and NW that should be answered in the future studies. However, NW and RW artificially infected with the similar doses of the microsporidian spores should be used to avoid of increased variation in results from colony to colony, cage to cage, and experiment to experiment. Another issue is the ability of bee intestinal epithelial cells to respond to apoptotic processes. The inhibition of apoptosis was shown to be pivotal for a successful infection of *N. ceranae* [42]. Kurze et al. [43] showed that *N. ceranae* reduces apoptosis in sensitive honeybees by enhancing inhibitor of apoptosis protein-(iap)-2 gene transcription. Xing et al. [44] presented that *N. ceranae*-responsive genes are decreased in the infected midguts after a longer time from post-inoculation (dpi). The differentially expressed genes in workers’ midguts are associated with six cellular immune pathways and three humoral immune pathways. Since the reaction to stress caused by this pathogen is different in queens and workers [5], and rebels are more similar to queens in many anatomical and physiological features [9,10,11,12,32], it can be assumed that this is one of the reasons for their different tolerance to *Nosema*.

Lyko et al. [14] revealed that over 550 genes showed methylation differences between queens and workers when they generated workers with queen characteristics by silencing DNA methyltransferase 3. Kucharski et al. [45], Chen et al. [46], and Shi et al. [47] showed that queens have lower global DNA methylation level than workers, and DNA methylated genes such as genes involved in mTOR pathways can control queen–worker dimorphism. Our study provides the evidence that global DNA methylation in RW is significantly lower than in NW, particularly in 21-day-old bees, which also expands our knowledge about RW. This finding compared with the previous ones that global DNA methylation increases with the worker age [19,20] allows us to conclude that RW age “epigenetically” slower than NW, what is also in line with results of Cardoso-Júnior et al. [21] who expanded the worker lifespan by artificial genome demethylation. This is in line with our previous suggestions that RW are more queen-like and that the differences between RW and NW are determined epigenetically. Amarasinghe et al. [48] revealed the differences in the genome methylation between reproductive and non-reproductive queenless workers in *Bombus terrestris* as well. Moreover, the level of global DNA methylation in normal workers in our study is similar as in the publications by Strachecka et al. [19,20,49,50], Paleolog et al. [18], Schulz et al. [51]. Biostimulants and herbs reduce the level of global DNA methylation, while pesticides, acaricides, and other anthropogenic factors increase it at a certain age stage of bees [18,19,20,49,52,53,54]. In other organisms, i.e., *Osmia rufa* [55], *Varroa destructor* [56], human [57,58], the level of global DNA methylation is much higher than in *A. mellifera* workers. In turn, social hymenopterans, such as wasps, and ants, have low levels of DNA methylation [59,60,61]. Determining the level of global DNA methylation is one of the first steps in epigenetic analysis. The different DNA methylation analysis technologies are primarily based on the initial treatments of DNA samples: bisulfite conversion, endonuclease digestion, and others are the next steps [62,63,64,65]. Therefore, in this publication, we indicate epigenetic differences between reproductive and non-reproductive workers, which is an important clue for further research on honeybees, and above all, their phenotypic plasticity.

## 5. Conclusions

RW in this study appeared to be the queen-like workers. They lived longer than NW and their presence expanded longevity of NW in a similar way as the presence of a queen, but to a much lesser extent. This expands possibilities of the use of honeybees as a model for studies of senescence. We also suggest mutual interactions between NW and RW affecting their lifespans as a subject of future studies.

RW, similarly to queens, seemed to be more nosema-tolerant, as well as queen-like when one considers this aspect of the link between longevity and body maintenance. However, RW coped better with the nosemosis during its course, as they lived longer than NW despite the fact that they exceeded them with the spore load at the same time. Therefore, surprisingly, these tolerant bees seemed to be more sensitive to the spore penetration. This needs clarification in future studies not only to better understand the mechanisms of the nosema tolerance but also to expand our knowledge about multifactorial mechanisms shaping tradeoffs between body maintenance and longevity in social insects, which have been intensively studied recently [5,30,66].

Differences between queen-like RW and NW had to have the epigenetic background, as global DNA methylation differed between those two worker types, particularly when impact of their ageing was considered. Hence, RW might be also “epigenetically” queen-like. Although honeybees have already been proposed as a useful model for epigenetic studies [17,67], epigenetic differences between RW and both queens and NW, which we have revealed, make honeybees a more useful model for this research. This expanding of knowledge on RW, suggesting putting them “between queens and workers”, creates new possibilities for studies on mechanisms of the evolutionary success of the social insects as well. This is particularly important, as DNA methylation has been proved to be involved in the caste phenotypic plasticity and shaping the behavior of worker subcastes [15,68,69].

## Data Availability

The datasets generated during and/or analyzed during the current study are available from the corresponding author on reasonable request.

## Supplementary Materials

The following are available online at https://www.mdpi.com/article/10.3390/insects12111021/s1, Table S1: Auxiliary statistics helpful for interpretation of the data which concern the *Nosema* spore load [spore number] and comparison of graphs in Figure 3A and Figure 2B–D. Table S2: Auxiliary statistics helpful for interpretation of data in Figure 4A,B; differences between RW and NW.
