# Supplementary material for: Reproductive Potential Impacts Body Maintenance Parameters and Global DNA Methylation in Honeybee Workers (Apis mellifera L.)
Source: Insects. 2021 Nov 12;12(11):1021. doi: 10.3390/insects12111021 (PMC8617817; doi:10.3390/insects12111021)
Supplement: Supplementary file 1 [file insects-12-01021-s001.zip › insects-1425425-supplementary.pdf]

Supplementary Materials

Table S1. Auxiliary statistics helpful for interpretation of the data which concern the *Nosema* spore load [spore number] and comparison of graphs in Figure 3A, 2B, 2C, 2D.

Explanations: RW and NW – respectively rebel workers and normal workers. day 9, day 11, day 15, day 17, day 19 – statistical interpretation of differences between RW and NW on the e.g. 9th, 11th, 15th, 17st , and 19th day of the worker life. Read typed – insignificant.

Environment		Phenotype	Mean	SD	F	p
Repetition 1 NW and RW are mixed within each cage	Day 9	RW	10.60	5.8356	0.04217	0.83780
		NW	10.37	4.5930		
	day 11	RW	16.79	6.0496	4.7911	0.03138
		NW	14.23	4.7047		
	Day 15	RW	27.65	6.2673	24.569	0.00000
		NW	21.90	4.2973		
	Day 17	RW	32.37	6.2679	37.496	0.00000
		NW	25.34	4.1569		
	Day 19	RW	36.18	6.1033	41.911	0.00000
		NW	28.60	4.6605		
Repetition 1 NW and RW were kept separately within different cages	day 7	RW	3.92	4.6271	0.08704	0.07704
		NW	4.50	5.4596		
	day 11	RW	13.07	6.0753	4.4292	0.04555
		NW	17.21	4.0034		
	Day 15	RW	24.84	5.9139	0.78150	0.38511
		NW	26.71	5.0600		
	Day 17	RW	30.23	6.8817	0.10016	0.75426
		NW	30.92	4.3934		
Repetition 1 hives	day 7	RW	0.00			
		NW	0.00			
	day 9	RW	5.66	6.6583	27.112	0.00649
		NW	32.66	6.0277		
	Day 17	RW	27.33	2.5166	64.059	0.00132
		NW	49.33	4.0414		
	Day 33	RW	55.66	15.3948	6.5146	0.05005
		NW	82.00	9.0737		
	Day 45	RW	74.66	21.3853	3.2848	0.14415
		NW	100.33	12.0138		
Repetition 2 NW and RW are mixed within each cage	day 7	RW	0.00			
		NW	0.00			
	Day 9	RW	0.00		1.6275	0.21253
		NW	1.13	3.4406		
	Day 11	RW	3.40	7.2571	16.626	0.00034
		NW	11.33	2.0283		
	Day 17	RW	19.20	7.8540	18.453	0.00019
		NW	29.40	4.7839		
	Day 31	RW	41.73	9.1936	14.458	0.00071
		NW	52.66	6.2845		
Repetition 2	Day 7	RW	0.13	0.5163	5.4935	0.02641
		NW	1.40	2.0283		

NW and RW were kept separately within different cages	Day 9	RW	2.66	4.4347	5.3094	0.02884
		NW	6.80	5.3478		
	Day 15	RW	19.93	12.360	1.0191	0.32138
		NW	23.46	5.5660		
	Day 17	RW	26.33	11.1333	0.20115	0.65725
		NW	27.73	4.7126		
	Day 23	RW	39.46	9.4556	0.24335	0.62564
		NW	40.80	4.4912		
	Day 29	RW	53.53	9.2339	0.20000	0.65816
		NW	52.26	5.9217		
	Day 33	RW	60.33	8.2779	1.1207	0.29881
		NW	57.53	6.0340		
Repetition 2 hives	Day 7	RW	15.00	9.4162	2.5881	0.15880
		NW	6.50	4.7958		
	Day 17	RW	41.50	7.5938	5.0888	0.06492
		NW	31.25	4.9916		
	Day 29	RW	65.50	9.1104	3.8278	0.09817
		NW	48.50	14.7986		
	Day 35	RW	77.25	12.3389	3.9452	0.09420
		NW	59.75	12.5797		
	Day 37	RW	82.25	9.5000	4.3059	0.08330
		NW	63.50	15.3731		
	Day 41	RW	89.00	8.67947	2.5080	0.17412
		NW	71.33	20.5020		

Table S2. Auxiliary statistics helpful for interpretation of data in Figure 4A, 4B; differences between RW and NW.

Explanations: RW and NW – respectively rebel workers and normal workers. Ovariole no. – number of the worker ovarioles on average. DNA metyl. (%) – percentage of the global DNA methylation (methylated cytosines) on average. DNA metyl. weight – weight of the methylated DNA cytosines [ng]

Trait and environment		Phenotype	Mean	SD	F	p
Ovariole no._cages RW and NW mixed in a cage		RW	13.20833	0.041074	21224.00	0.0000
		NW	4.74776	0.041057		
Ovariole no._cages RW and NW in a separate cages		RW	12.35893	0.056625	9595.20	0.0000
		NW	4.67549	0.054279		
Ovariole_3_hives_1-day	Ovariole no.	RW	11.90000	0.297727	211.35	0.0000
		NW	5.48000	0.326144		
	DNA metyl. (%)	RW	10.19000	0.125247	124.06	0.0000
		NW	8.12080	0.137202		
	DNA metyl. weight	RW	6.46433	0.036331	407.89	0.0000
		NW	5.37600	0.039799		
Ovariole_3_hives_7-day	Ovariole no.	RW	11.93333	0.249671	327.69	0.0000
		NW	5.42857	0.258434		
	DNA metyl.	RW	12.30500	0.015546	3066.10	0.0000

	(%)	NW	13.54393	0.016092		
	DNA metyl. weight	RW	7.26333	0.026139	630.81	0.0000
		NW	8.20821	0.027057		
Ovariole_3_hives_14-day	Ovariole no.	RW	12.17241	0.245144	388.07	0.0000
		NW	5.40000	0.241024		
	DNA metyl. (%)	RW	13.04897	0.286634	46.465	0.0000
		NW	15.78900	0.281816		
	DNA metyl. weight	RW	10.33172	0.122706	258.48	0.0000
		NW	13.09833	0.120644		
Ovariole_3_hives_21-day	Ovariole no.	RW	12.16667	0.247207	400.91	0.0000
		NW	5.16667	0.247207		
	DNA metyl. (%)	RW	16.41900	0.063110	45281.00	0.0000
		NW	35.41100	0.063110		
	DNA metyl. weight	RW	12.36033	0.014571	21440.00	0.0000
		NW	15.37767	0.014571		
